# Stent-Mediated Redistribution of Cerebral Venous Outflow in the Treatment of Severe Intractable Headache: A Case Report

**Published:** 2014-04

**Authors:** Nicholas Higgins, Frances Hall, John Pickard

**Affiliations:** * Addenbrooke’s Hospital, Cambridge, UK; † Addenbrooke’s Hospital, Cambridge, UK; ‡ Addenbrooke’s Hospital, Cambridge, UK

**Keywords:** Headache, epistaxis, cerebral venous outflow, venous sinus anatomy, venous sinus stenting

## Abstract

We describe a patient with multiple symptoms but whose primary complaint was of headache, in whom no firm diagnosis was made in two years, who was resistant to all treatment, until a markedly asymmetrical cranial venous outflow came to be regarded, not as normal variant anatomy but as fundamental to the clinical problem. Deliberately altering this anatomy in favour of a more symmetrical arrangement by stenting a hypoplastic transverse sinus brought about an immediate, profound and sustained clinical improvement. This result challenges the existing consensus on what is acceptable as normal in respect of cranial venous outflow. It raises intriguing questions about the relationship between neurological symptoms and the vagaries of cranial venous outflow anatomy. It suggests there may be new opportunities in the investigation of chronic headache.

## Introduction

Short of venous sinus thrombosis, the suggestion that neurological symptoms or syndromes might be attributed solely to venous disease is generally met with scepticism or outright disbelief.^1,2,3^ Yet, if cerebral venous outflow obstruction from venous sinus thrombosis can produce a florid and sometimes fatal neurological illness, there is no reason, in theory, why lesser degrees of venous obstruction should not produce a more subtle neurological disorder. What is more, given the complexity of the clinical syndrome that may result from frank venous sinus thrombosis,^4^ the clinical correlate of a lesser degree of venous obstruction might not be easy to recognise. Add to this a wide acceptance of almost any appearance of the venous sinuses and neck veins as part of normal anatomical variation,^5,6,7,8^ then attributing symptoms to a particular venous anatomical arrangement becomes almost impossible.

Against this background, headache clinics are overwhelmed with patients whose diagnoses are either unknown or comprise little more than a description of their symptom complex with no pathological substrate; this usually confirmed by normal blood tests and normal radiology.^9,10^ Could any of these patients have venous disease and how would you prove it if they did? We describe a patient with severe debilitating headache and subjective neurological symptoms who was undiagnosed for two years until investigations were specifically targeted at the cerebral venous circulation. Treatment resulted in a profound clinical improvement, which has been sustained over more than two years of follow-up.

## Case report

### Initial history and investigations

A 33-year-old woman was referred with headache and multiple symptoms, having already been extensively investigated the by neurology services at several other institutions.

She gave a history of being completely well until she had been woken from sleep 18 months earlier with severe bilateral retro-orbital headache, at first associated with nausea and vomiting. There had been no head injury, alcohol binge or any other obvious precipitant. This headache had been present constantly since its first appearance and had been unresponsive to ordinary analgesics, pizotifen, amytriptyline, topiramate and labetolol. Opiate patches provided some relief.

Two months after the onset of headache she had begun to complain of visual disturbance; at first, a dark spot in the inferotemporal field of the left eye, eventually progressing to a substantial bitemporal hemianopia. At the same time, she had noticed variable numbness in her left face, arm and lower leg with occasional similar symptoms on the right.

**Figure 1:**
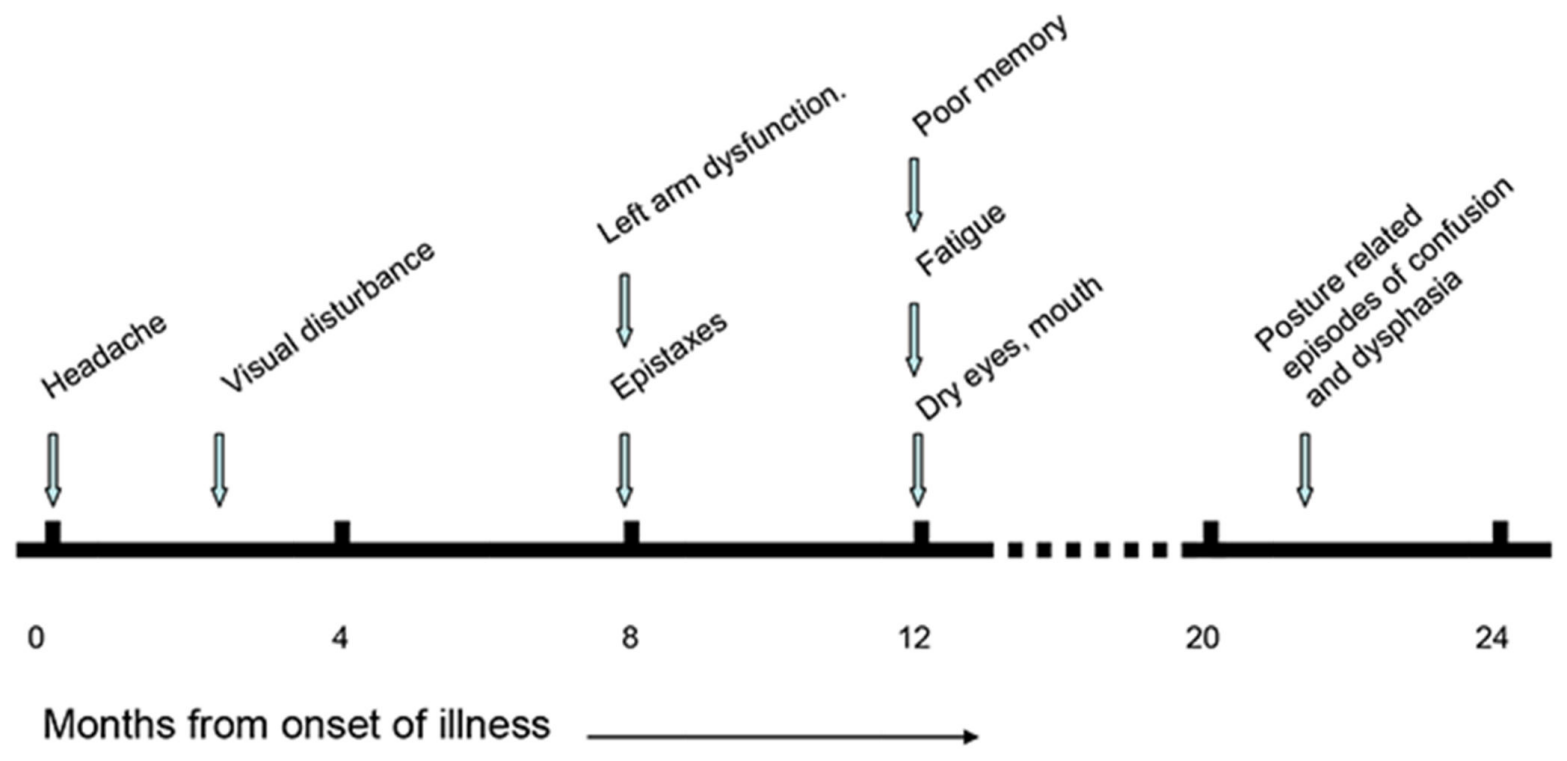
Timeline of symptom development

Eight months after the onset of headache she had begun to experience frequent and recurrent nose bleeds. Sometimes, these were preceded by a reddish discoloration over her left cheek and they were followed by a period of confusion, dizziness and difficulty speaking. Around the same time, she had noticed mild weakness in her left arm, which meant she could not hold up a cup or kettle, or use a pen on that side (she was normally ambidextrous). Twelve months into her illness she noticed that her periods had become irregular; her hair growth had slowed; and she was suffering from poor memory, fatigue, dry skin, dry eyes, dry mouth and dry nose. (Figure 1).

On examination, she was slim and looked well. She was mildly hypertensive when first seen but normotensive subsequently. She exhibited a mild Raynaud’s phenomenon and a subtle livedo reticularis rash over her knees and ankles but there were no other significant non-neurological findings. Her optic fundi were normal. Visual fields to confrontation at first appeared to confirm a bitemporal hemianopia but there were inconsistencies in her response to testing that cast doubt on this finding. Other cranial nerves were normal. There was a mild, non-pyramidal weakness of the left arm with no accompanying upper motor neuron signs. There was impairment to light touch up to the left ankle and throughout the left arm to the shoulder.

**Figure 2:**
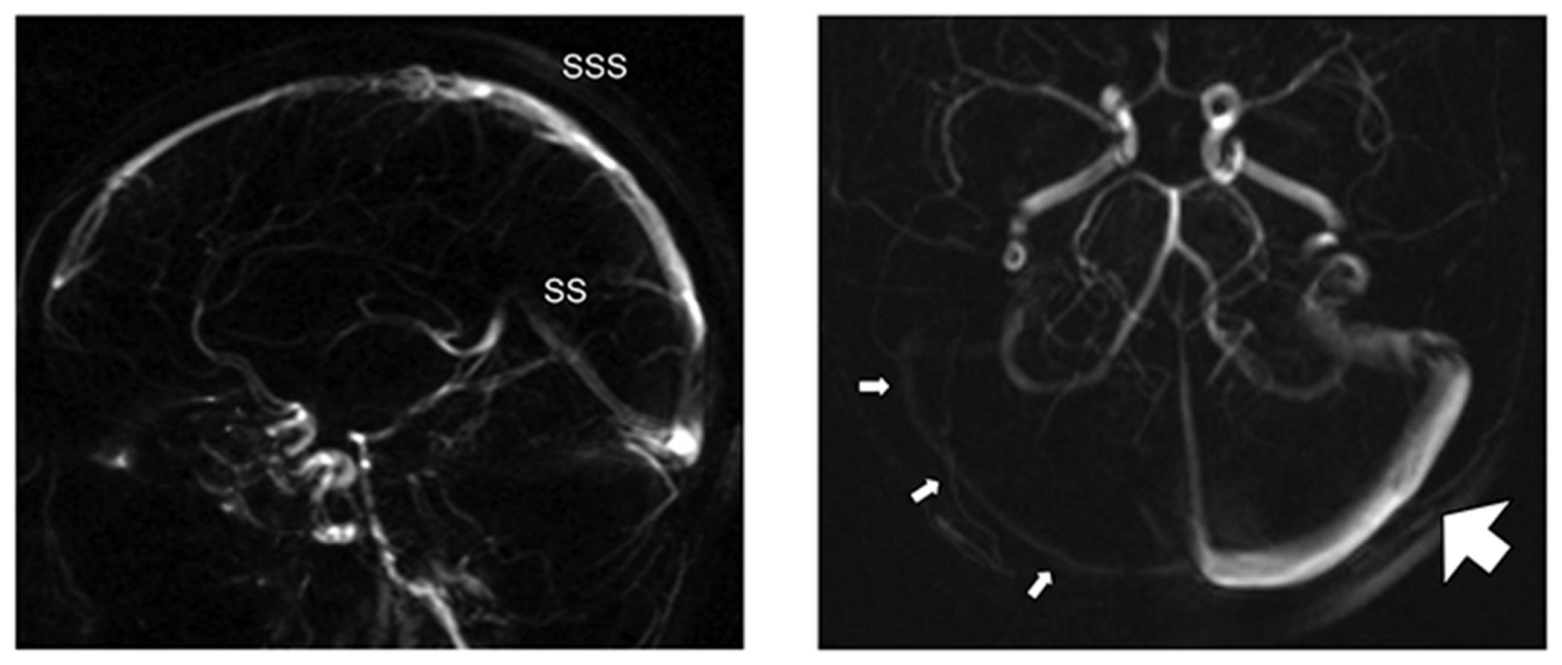
Magnetic resonance venograms (a) Left-hand image gives a lateral view of a normal superior sagittal sinus (SSS) and straight sinus (SS). (b) Right-hand image gives an axial view through the posterior fossa showing marked dominance of the left transverse sinus (large arrow). The right transverse sinus is patent but small (little arrows). (There is some contamination of both images by signal from the carotid and vertebrobasilar systems.)

Brain CT had been normal. Brain MRI had been normal except for a 7 mm nodule in the right pituitary. This nodule had minimal impact on pituitary volume and was unchanged over a four-year period. There was no suprasellar extension and no compression of the optic chiasm. MR venography had been unremarkable, though showing marked dominance of the left transverse sinus (Figure 2). Blood haematology and biochemistry had been normal. Full endocrine testing had been unremarkable, except for intermittently positive antinuclear antibodies. CSF pressure was unremarkable and biochemistry had been normal. Visual evoked responses and multifocal electroretinography had been normal. Specialist ear, nose and throat evaluation, including nasal endoscopy, had not established a cause of her epistaxis.

No neurological diagnosis was reached. A rheumatology opinion was sought and the possibility of a connective tissue disorder discussed, with primary Sjogren’s the most likely contender. However, there was a consensus that a unifying diagnosis which would account for the whole clinical picture was unlikely to be found, leaving symptomatic treatment the only therapy on offer.

### Investigation of cerebral venous outflow

**Figure 3:**
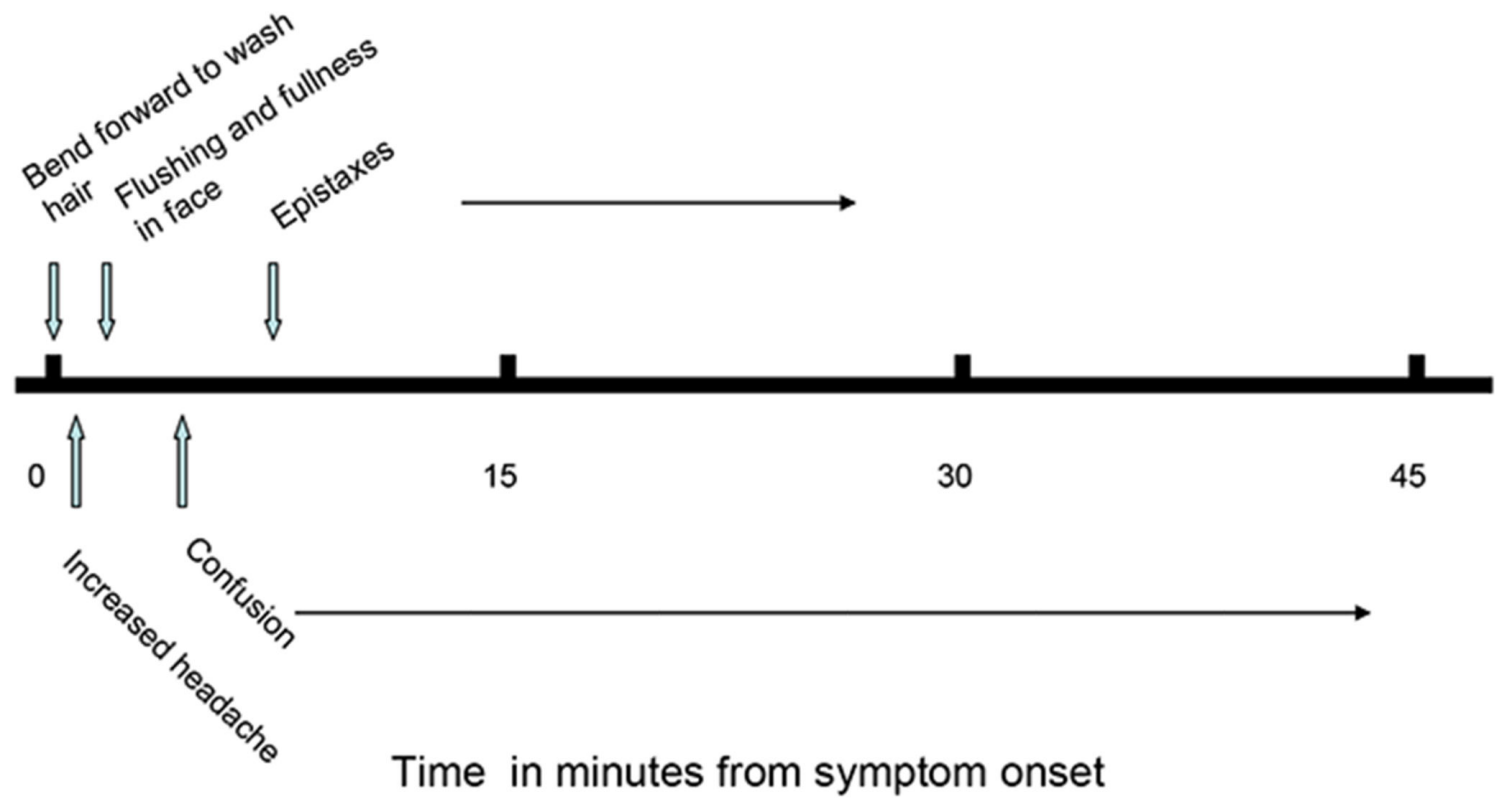
Timeline of a typical episode of epistaxis and confusion

Her symptoms progressed, especially epistaxes, which were now occurring several times a week and often formed part of a stereotyped response to a particular posture. Bending forwards to wash her hair, for example, markedly exacerbated her headache. This was often followed by flushing and fullness in her face, which was usually followed, in turn, by brisk epistaxis lasting up to 20 minutes. At the same time, whether or not she developed epistaxis, she would often experience a period of confusion, dizziness and difficulty with speech lasting about 45 minutes. She was unable to work (Figure 3).

About this time she reported an intermittent rushing noise on the left side of her head, more noticeable when she lay on her left side. There was no audible bruit but to exclude an underlying vascular cause for her symptoms she was referred for catheter angiography.

**Figure 4:**
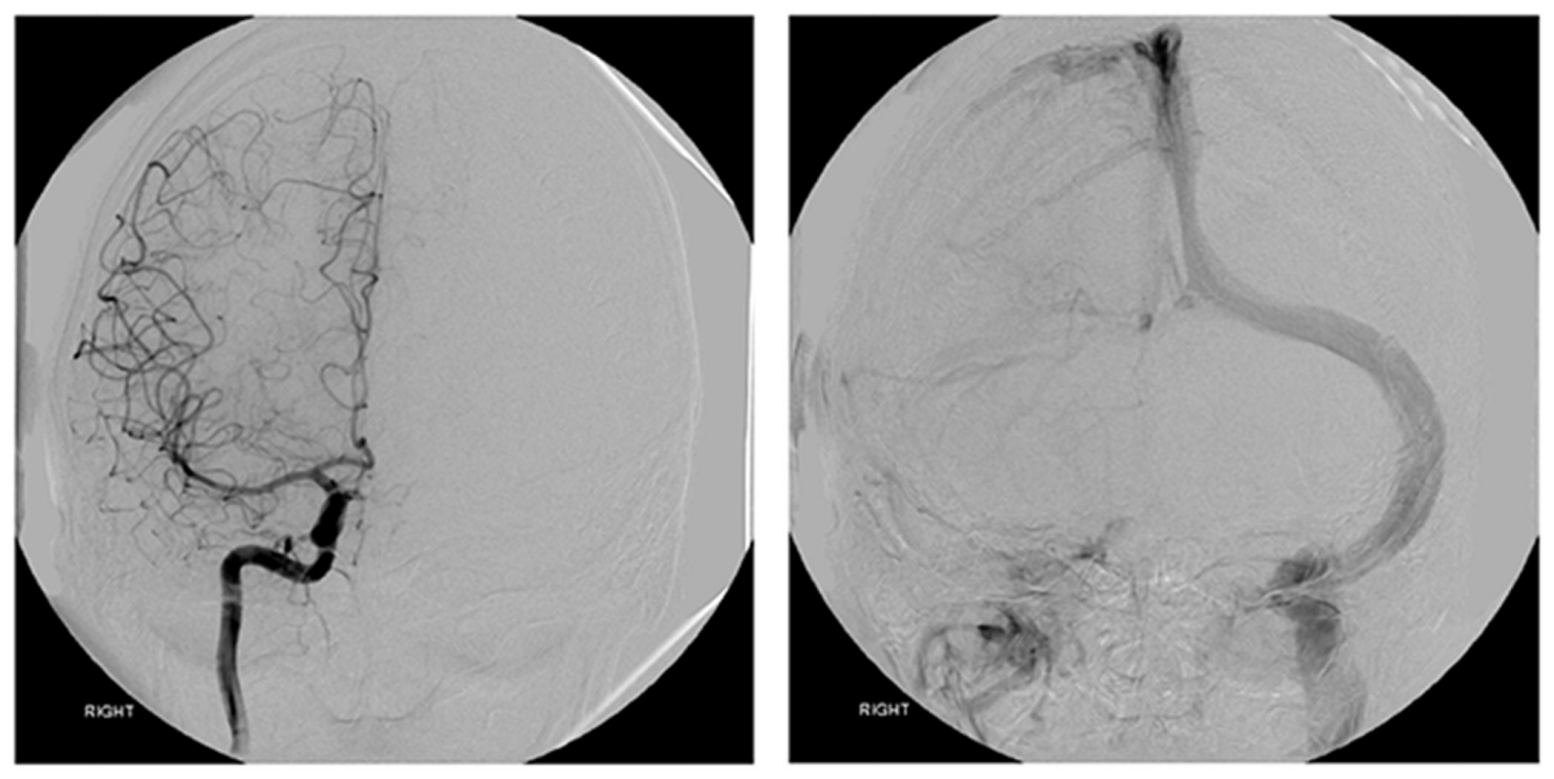
Right carotid angiogram (frontal view) The arterial phase (left image). The venous phase (right image) shows venous outflow almost exclusively to the left side.

**Figure 5:**
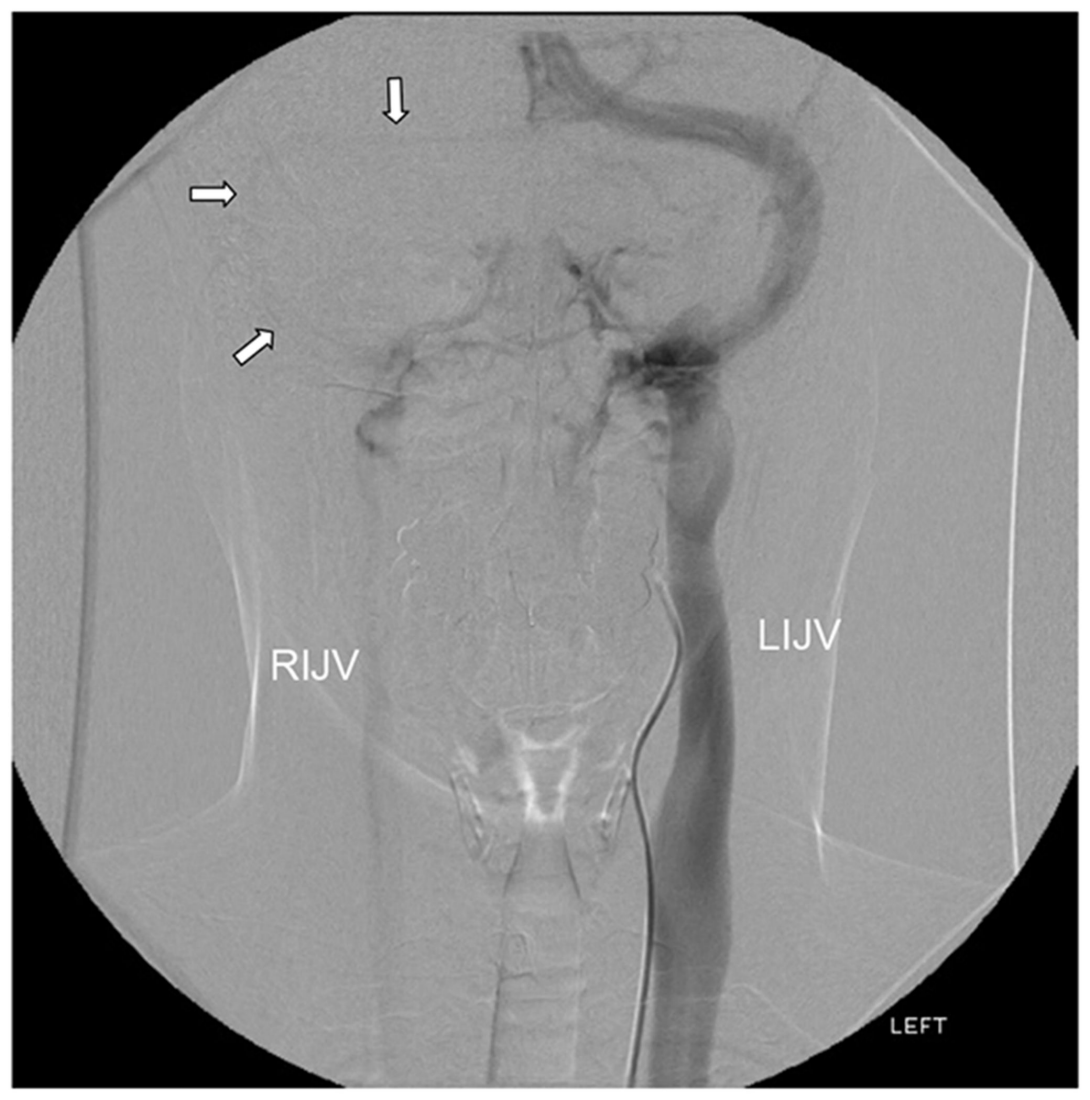
Right carotid angiogram (frontal view, centred over skull base and neck) The left internal jugular is normal (LIJV). The right internal jugular vein is small (RIJV). There is faint opacification of small right transverse and sigmoid sinuses (arrows).

The angiogram showed normal arterial anatomy. Venous anatomy was also within accepted normal limits, although there was very marked dominance of the left transverse sinus which took the bulk of venous outflow from the sagittal and straight sinuses (Figure 4). The left jugular vein was also very much larger than the right (Figure 5).

Looking to test the significance or her particular venous anatomy, gentle manual compression of the right side of the neck produced no response. Gentle compression of the left side, however, caused her face to become suffused with the colour puce. This receded immediately compression was released but was followed by a period of mild confusion and disorientation, difficulty with speech and left facial numbness which lasted about 45 minutes. Once she had recovered she was able to attest to the similarity of this episode with those she had been experiencing at home.

**Figure 6:**
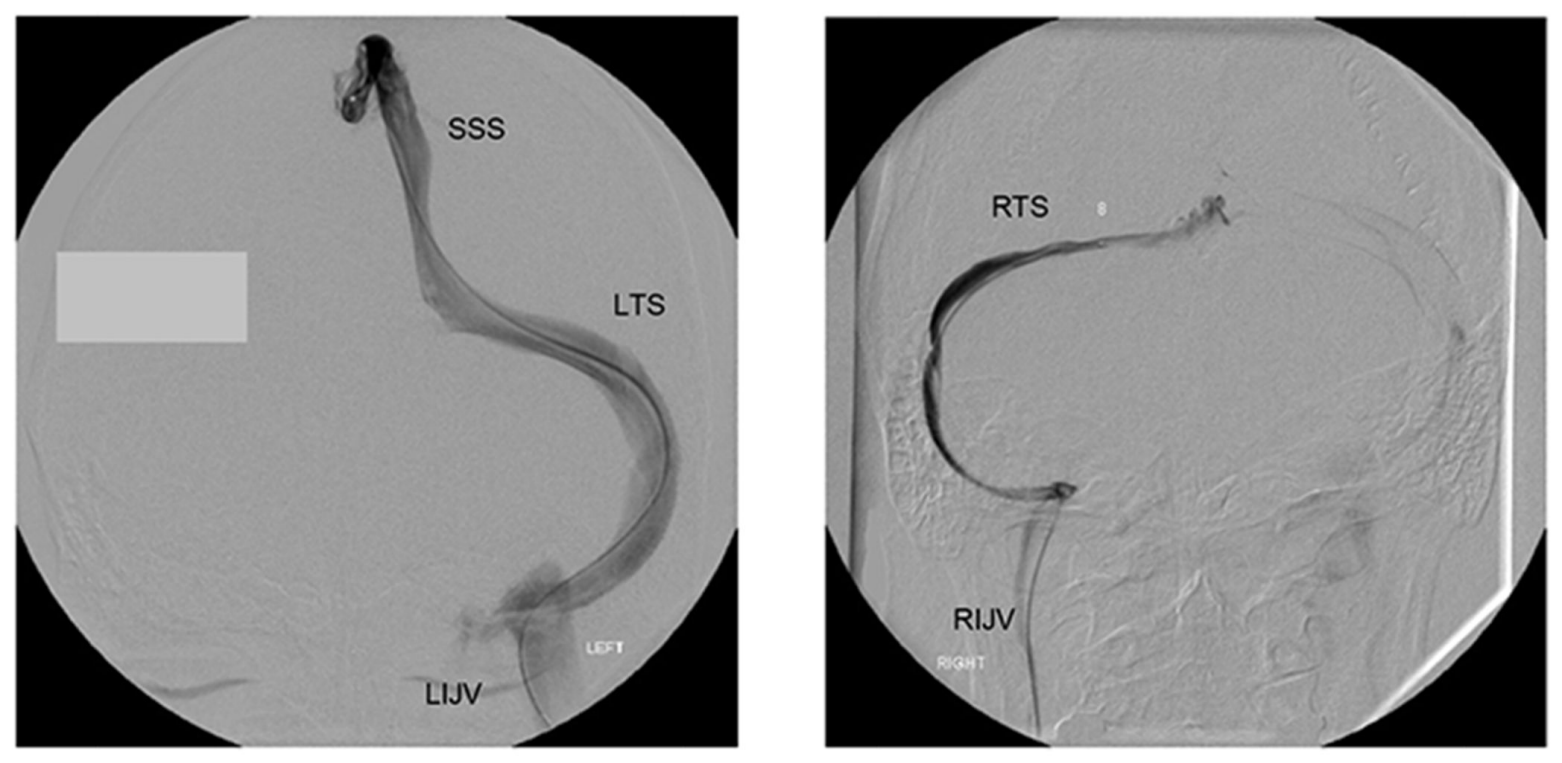
Catheter venogram, frontal view Left image shows radiographic contrast injected through a catheter in the sagittal sinus (SSS) draining exclusively into the left transverse sinus (LTS) and then the left internal jugular vein (LIJV). Right image shows a contrast injection from a catheter positioned in the right transverse sinus (RTS): the right transverse and sigmoid sinuses are small, as is the right internal jugular vein (RIJV).

She was taken back into the angiography suite for catheter venography. This procedure, performed under local anaesthesia, involves examination of the venous sinuses and jugular veins using a catheter passed cranially from a common femoral puncture. Injection of radiographic contrast medium allows visualisation of the sinuses and the direction of flow in them. Attaching a transducer to the catheter hub allows the recording of intrasinus pressures (Figure 6).

**Figure 7:**
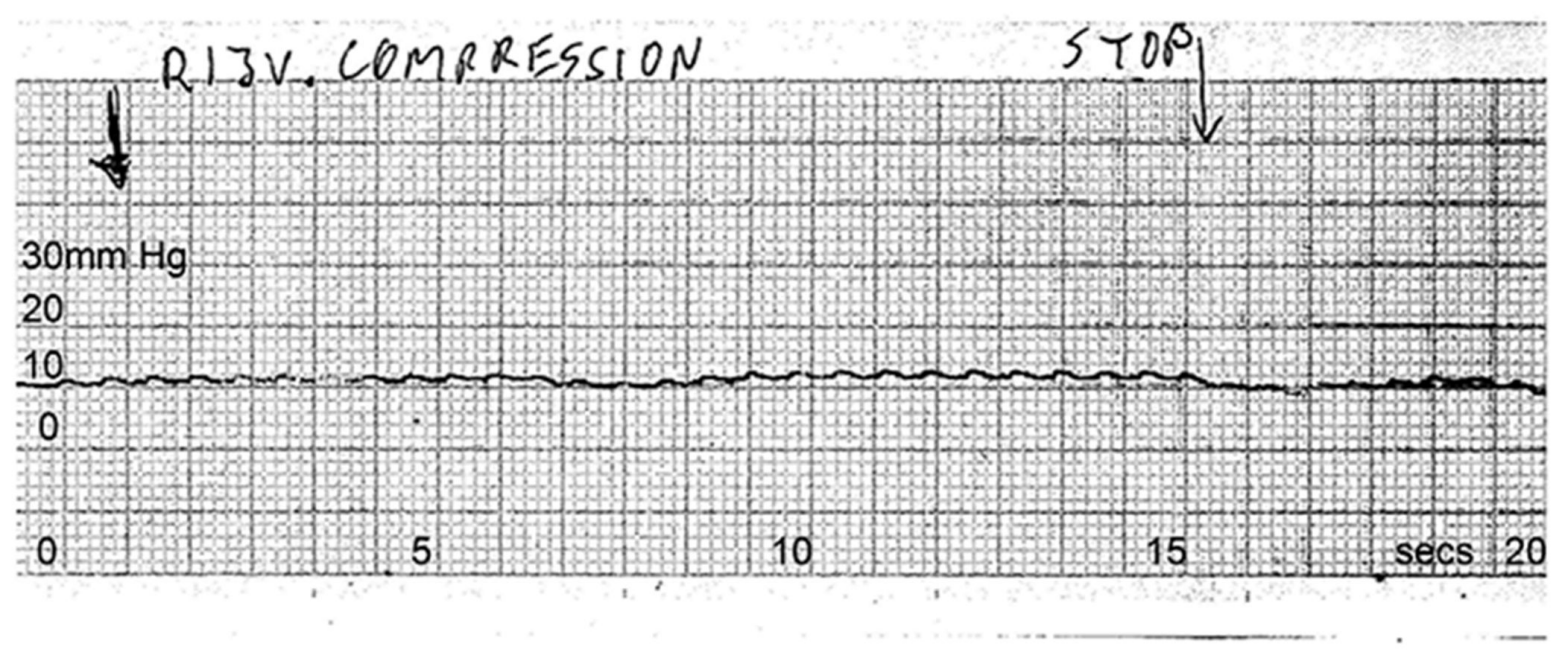
Continuous pressure recording from the sagittal sinus during right jugular compression Drawn arrow marks beginning of right jugular compression lasting 15 seconds. Sagittal sinus pressure increases by about 2 mm Hg during that period, then returns to baseline once compression is released.

**Figure 8:**
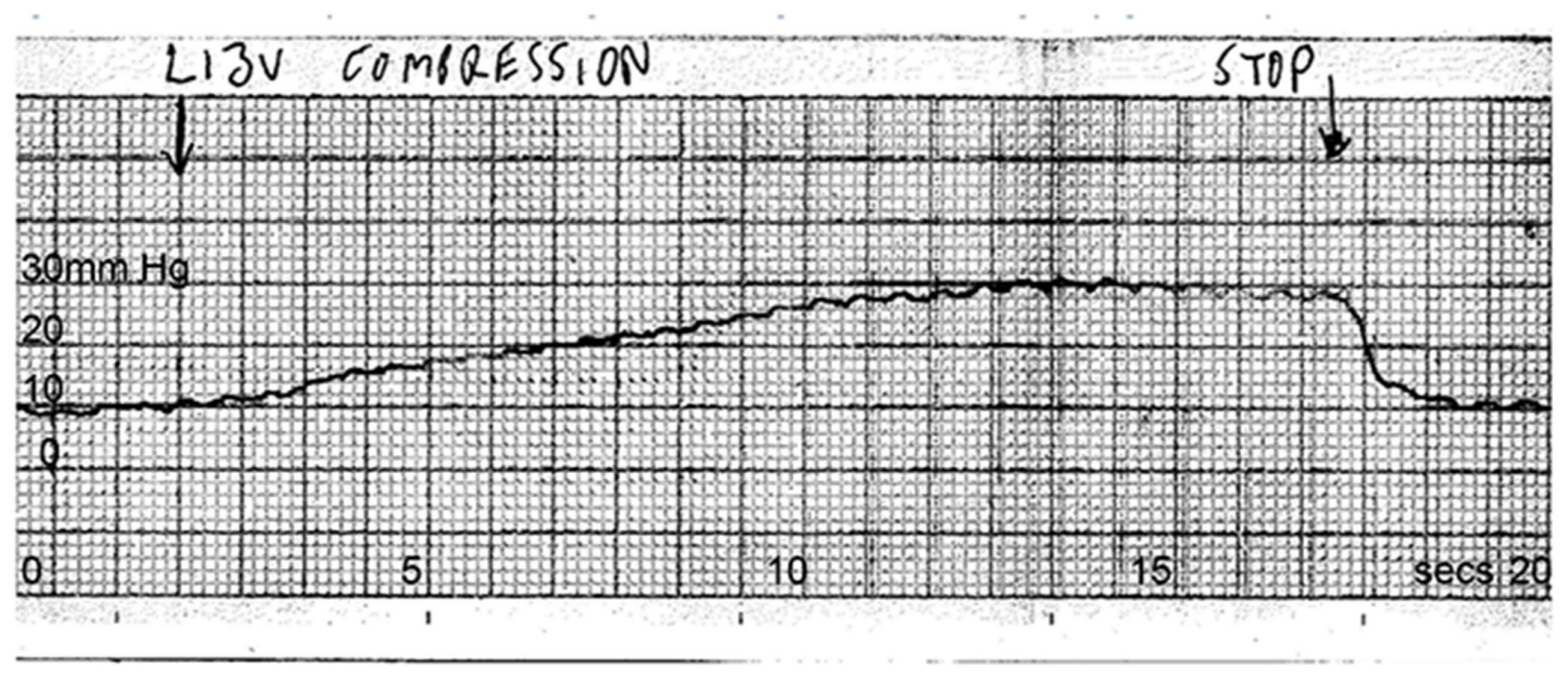
Continuous pressure recording from the sagittal sinus during left jugular compression Drawn arrow marks the beginning of left jugular compression lasting about 15 seconds. Sagittal sinus pressure increases gradually by 20 mm Hg during that period, then returns to baseline once compression is released.

Under baseline conditions, sagittal sinus outflow was exclusively to the left side; the right transverse sinus being patent but narrow and with minimal antegrade flow (Figure 6). Sagittal sinus pressure was recorded at baseline (9 mm Hg), then with right jugular compression (11 mm Hg), then with left jugular compression (29 mm Hg) (figures 7 and 8). Right atrial pressure was 5 mm Hg. This meant that a 24 mm Hg pressure gradient developed between the sagittal sinus and right atrium with left jugular compression. Further measurements showed that most of this gradient (14 mm Hg) was along the transverse sinus. Her symptoms after jugular compression developed as before but she was able to cooperate with the procedure and was fully recovered in about 45 minutes.

### Treatment by venous sinus stenting

By this time it was more than two years since the onset of symptoms and she had not worked for six months. Therefore, with no alternative diagnosis on offer, we elected to proceed on the basis that her clinical syndrome was a consequence of the observed asymmetry in cranial venous outflow. There was no evidence of previous sinus thrombosis and the right transverse sinus was presumed congenitally hypoplastic. This meant there was no indication of what might have precipitated the onset of symptoms. Nevertheless, the hypothesis for her current condition was that she was so highly dependent on left-side cranial venous outflow that whenever the left jugular vein was compressed, she developed symptoms of venous compromise and raised intracranial pressure, of which headache, confusion and disorientation were one expression and epistaxis another.

She was booked for stenting of the right transverse sinus with the aim of facilitating an alternative pathway for cranial venous outflow whenever flow in the left jugular vein was compromised. The right transverse sinus was small but still provided a potential conduit for venous outflow from the sagittal sinus in these circumstances, although currently only at the cost of a substantial pressure gradient. Stenting would enlarge this conduit to allow a greater flow of blood with a smaller pressure gradient under the same conditions.

**Figure 9:**
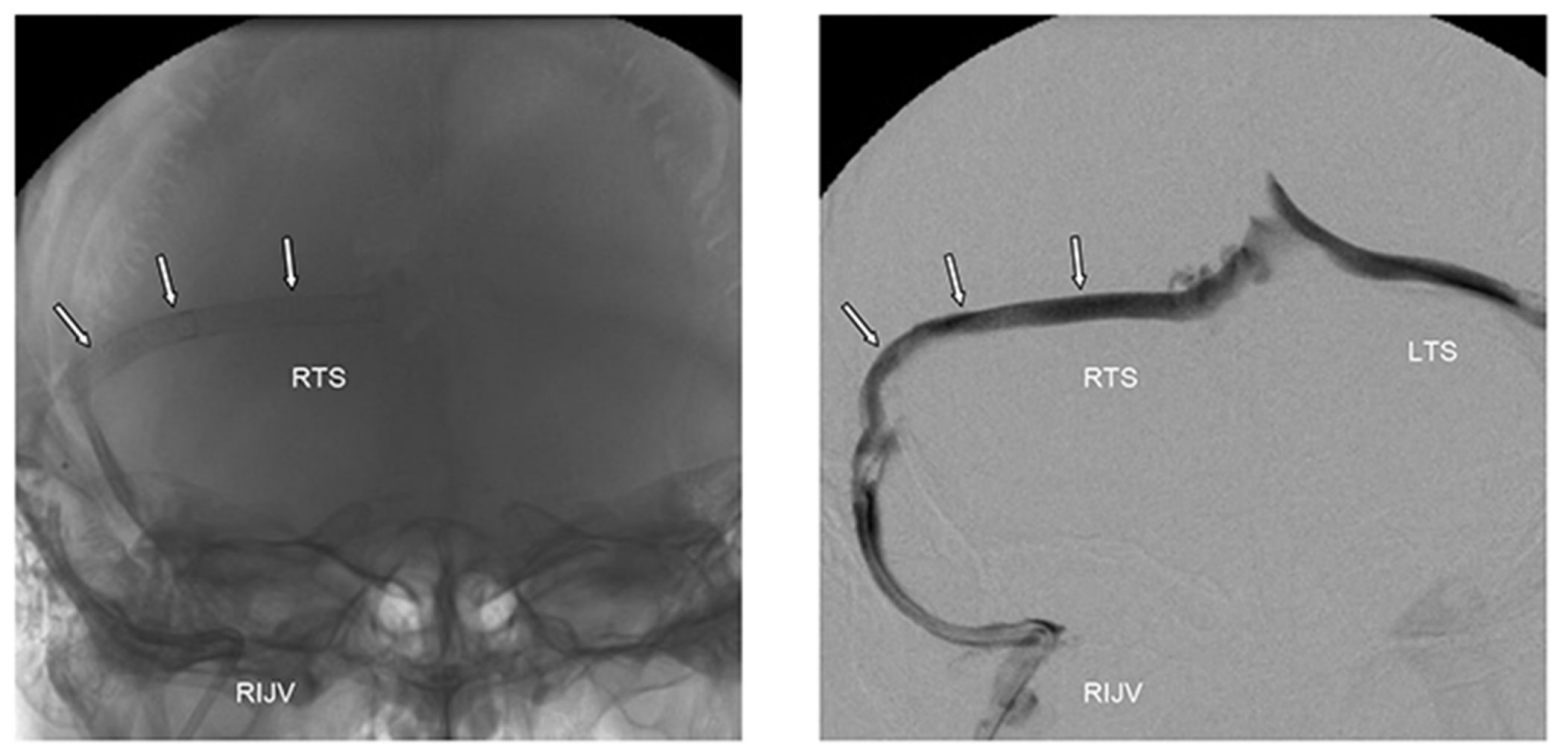
Frontal view venogram after stent placement in right transverse sinus Left image: unsubtracted film showing the stents along the right transverse sinus (arrows). The stent delivery catheter can be seen entering the sigmoid sinus from the right internal jugular vein. Right image: subtracted view in same projection following injection of the sinus with radiographic contrast. There is antegrade flow from the (stented) right transverse sinus (RTS) into the right jugular vein (RIJV) and retrograde flow across the confluence of sinuses into the left transverse sinus (LTS).

Stenting was performed under general anaesthesia using a right jugular puncture. In the procedure a 5F guide catheter was passed into the right sigmoid sinus under X-ray control and a guide wire passed across the right transverse sinus into the sagittal sinus. This was used to deploy 4 mm diameter stents (Pharos; Micrus Corp) along the whole of the right transverse sinus under heparin anticoagulation (Figure 9).

She had some headache localised to the site of the stents the next day but, otherwise, she described her head as feeling better than it had done at any time since she had become ill. Under supervision she bent forwards, in the same way that would previously have produced an immediate exacerbation of symptoms, without incident. She was discharged with instructions to take aspirin 75 mg and clopidogrel 75 mg daily for three months as a precaution against thromboembolic complications.

At six-week follow-up she said that her quality of life was greatly improved. Right-side headache (from the stents) had resolved. She still had some generalised headache but this was much less severe than before stenting. Bending forwards still made her headache worse, although not as dramatically as before. She had had no further epistaxes and no further episodes of confusion. Normal function had returned to her left arm.

Repeat catheter venography showed baseline pressure in the sagittal sinus similar to that previously (11 mm Hg). This increased to 19 mmHg with left jugular compression; that is much less than before and now not accompanied by any change in her clinical status. Pressure measurements undertaken in the right lateral sinus during left jugular compression (diverting most cranial venous outflow through the right side) now showed only a 1 mm Hg gradient along the stented right transverse sinus but there remained a 4 mm Hg gradient along the right sigmoid sinus.

**Figure 10:**
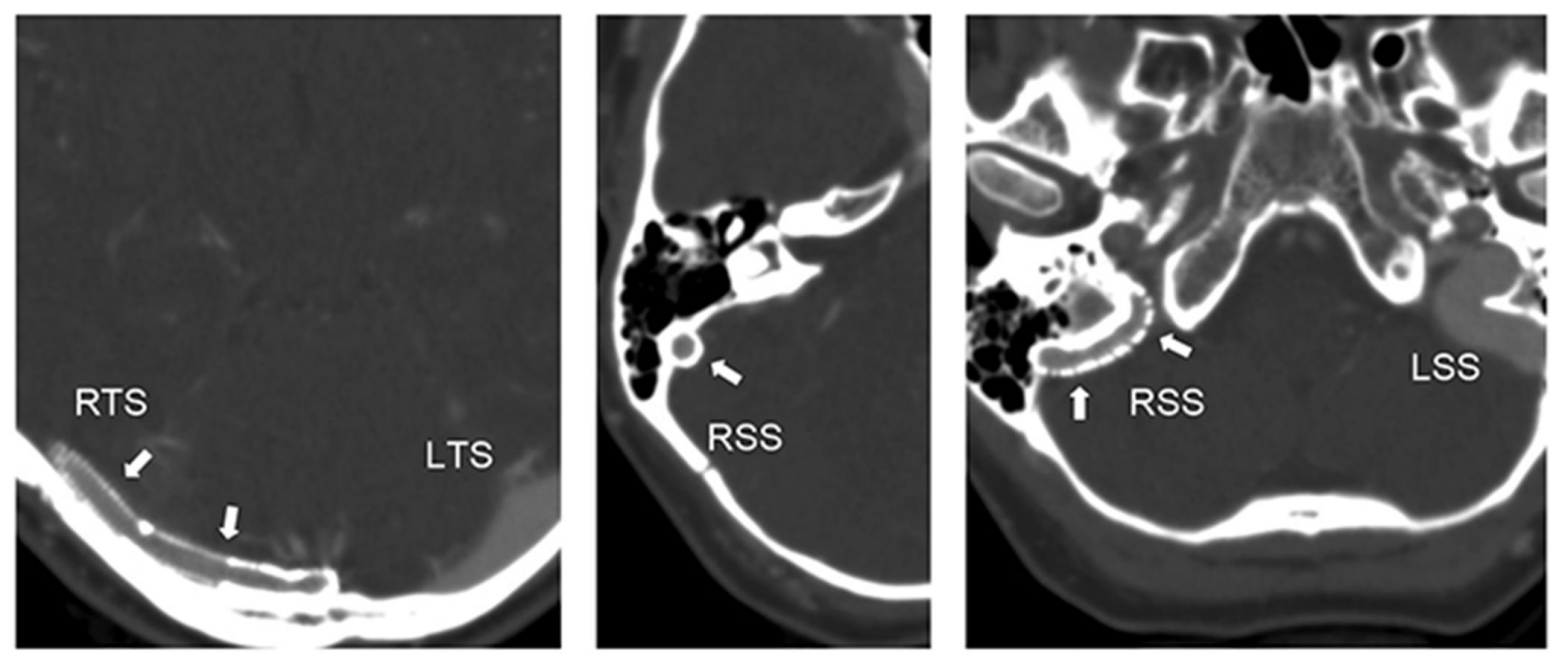
Follow-up CT venogram of the posterior fossa Axial scans at different levels showing contrast opacification inside the stents (arrows). Left-hand image shows stents in the right transverse sinus (RTS), middle image in the right sigmoid sinus (RSS) and right-hand image at right sigmoid sinus outflow into the jugular vein. Note the normal transverse and sigmoid sinuses sinuses on the left side (LTS, LSS).

She was admitted for a second stenting procedure, this time of the rest of the right lateral sinus, effectively extending the previous stents through the right sigmoid sinus into the jugular bulb. This produced a further improvement in headaches. She had all the stents dilated up with a balloon in a third procedure, after which her headache virtually resolved. Subsequent CT and catheter venography (13 months after the first stenting procedure) showed all the stents widely patent (Figure 10) and sagittal sinus pressure increasing by only 4 mm Hg in response to left jugular compression.

In two years of follow-up there have been no further epistaxes and no further episodes of confusion. She still complains of restricted vision but said there had been some improvement since the start of treatment (her visual fields never having been recorded as abnormal on formal testing). Her memory had returned to normal. She still suffers with dry eyes, mouth and nose. Her periods are regular but short. She is still fatigued but this is less disabling than previously. She is back at work full time. She is off all medication.

## Discussion

With regard to chronic headache, clinicians instigate scans in the strong expectation that the results will be normal and radiologists view the images on the understanding that symptoms rarely have any basis in a structural lesion.^9,11^ The exceptions (tumour and hydrocephalus, for example) are usually not difficult to detect.

This understanding between these two medical disciplines may seem entirely appropriate at first sight. However, a critical look at the way in which radiologists arrive at a diagnosis of normality when reviewing a brain scan suggests that a scan declared as normal in these circumstances, in reality, brings little independent evidence to the clinical diagnosis.

Thus, it is probably self evident that radiologists develop much of their expertise by reviewing large numbers of images, both normal and abnormal. This allows the accumulation of a mental database of normal appearances for use as a reference when differentiating significant pathology from what is irrelevant and what is normal variant anatomy. Studies on normal volunteers are available^12,13,14^ but in practice this database is largely derived from patients whose scan findings, whatever they might be, are assumed to be unrelated to the illness under investigation. This might be reasonable in the case of a patient who is asymptomatic and has a brain scan looking for metastatic cancer, for example. Here, any appearance out of the ordinary which is not tumour could reasonably be ascribed as incidental or a variant of normal. However, it might not be reasonable to make the same deduction in the case of a patient complaining of a headache. In this instance, especially if there was no pathological diagnosis, how could you be sure that whatever you had seen on the scan was not contributing to symptoms? The question is pertinent because patients with headache contribute substantially to the workload of any radiology department and, therefore, substantially to this mental database of normal appearances.

With respect to the venous sinuses, this gives rise to a problem encapsulated in attempts to determine normal, in vivo anatomy. Ayanzen et al.^15^ performed MR venography in 100 patients whose MRI brain scan had already been diagnosed as normal, looking to catalogue the gamut of normal variant appearances for reference purposes. They found flow gaps in 31% of transverse sinuses, most of them in non-dominant sinuses, but a few in co-dominant sinuses, and argued that these should not be mistaken for sinus disease, a finding accepted largely without question by both clinicians and radiologists.

Taken for granted in this work, however, is that flow gaps in the venous sinuses could not possibly be responsible for symptoms. Yet, if one considers the commonest symptom and most frequent diagnosis in patients with a normal MRI scan – headache of unknown cause^16^ – then this assumption becomes highly questionable. Moreover, if one takes the trouble to recruit a control group which is headache-free then the presence of flow gaps in the venous sinuses takes on an entirely new and important significance. Higgins et al.,^17^ for example, compared the appearances of the venous sinuses in patients with idiopathic intracranial hypertension with those of normal volunteers specifically screened for the absence of headache. They found flow gaps in both transverse sinuses in more than 50% of patients with idiopathic intracranial hypertension but in none of the controls. Using ‘supernormal’ controls in this way, therefore, gave birth to a radiological sign of raised intracranial pressure (bilateral transverse sinus flow gaps) hitherto completely submerged by the incorporation of patients, whose symptoms should have excluded them, into the collective understanding of normative appearances.

On debatable grounds, therefore, radiologists are using patients with chronic headache, in whom the scan does not show any obvious structural abnormality, as their yardstick of what is acceptable as normal. They are encouraged in this view by clinicians who cite the frequency of a normal scan in such cases as evidence that most chronic headache does not have a basis in a structural lesion. What this means is that, without recognising it, radiologists and clinicians are colluding in a circular argument, effectively excluding common observations from being implicated in a common complaint.

The same concerns apply to the concept that asymmetry is a normal condition of cranial venous outflow. This idea has been developed over many years of cerebral angiography independently of CT or MRI. Yet, here as well, the observations on normal anatomy are derived from patients who are ill and have to be judged in that context. Much of angiography, for example, is undertaken in the investigation of headache with or without proven intracranial haemorrhage. Often, no cause of symptoms or haemorrhage is found and often there is marked asymmetry of cranial venous outflow through the transverse sinuses. This almost never invites comment and is usually accepted as part of normal anatomical variation.^18^ Yet, how can one be sure that the appearance of the venous sinuses in these cases is irrelevant? It is widely recognised, for example, that some cases of subarachnoid haemorrhage have a venous aetiology,^19^ reason enough by itself, to disqualify any of these patients from forming the basis of our understanding of normal venous anatomy.

More recent attempts to document the asymmetry or otherwise of venous sinus outflow have focused on MR venography, again with asymmetry, sometimes quite marked, a common finding.^6,7,8^ These studies all used the fact of a normal MRI as a licence to interpret any appearance of the venous sinuses as part of normal variant anatomy. Yet, MRI in all these studies was performed in the investigation of symptoms, not always specified, making their conclusions with respect to the venous anatomy equally suspect.

In the case we describe, no progress was made in diagnosis or treatment until the observed asymmetry of cranial venous outflow came to be viewed as part of the problem rather than as simply an expression of normal venous anatomy. The catalyst for this change of view was a somewhat speculative clinical test in which each of the internal jugular veins was compressed in turn and the result observed. This is not a manoeuvre described in the medical literature, except with reference to tinnitus in association with idiopathic intracranial hypertension.^20^ Nevertheless, its outcome here established a connection between symptoms and the particular venous anatomy. Then, having accepted this connection, partial restoration of outflow symmetry by stenting a hypoplastic transverse sinus brought about an immediate and profound symptomatic improvement. There were no further epistaxes, for example, despite instigation of dual antiplatelet treatment to maintain stent patency. Repeats of the jugular compression test no longer produced any adverse clinical symptoms. Subsequent procedures, in which the outflow asymmetry was further reduced, gave further incremental reductions in headache. There has been no deterioration in two years of follow-up.

Unexplained, of course, is what precipitated the clinical syndrome in the first place in a patient whose cerebral venous outflow anatomy appeared to be congenitally determined. Unexplained also is why headache was constant (though variable in intensity) when intracranial venous pressures and CSF pressure were normal in the resting state, although in this regard, the time course (45 minutes) of symptoms precipitated by a short period of left jugular vein compression is intriguing.

These questions aside, this case raises the possibility that asymmetrical cerebral venous drainage, particularly highly asymmetrical venous drainage, may be very unusual in the normal population. It suggests that, in the context of headache and unexplained symptoms, it may be inappropriate to write off such appearances as simply a manifestation of normal variant anatomy without clinical significance. It invites speculation that there may be an explanation in the venous anatomy for symptoms in other headache syndromes; although, without the insight that was afforded in this particular case by the jugular compression test, the diagnosis is unlikely to be easy.
